# Association of Polymorphisms in Plasminogen Activator Inhibitor-1 (*PAI-1*), Tissue Plasminogen Activator (*tPA*), and Renin (*REN*) with Recurrent Pregnancy Loss in Korean Women

**DOI:** 10.3390/jpm11121378

**Published:** 2021-12-16

**Authors:** Hee Young Cho, Han Sung Park, Eun Hee Ahn, Eun Ju Ko, Hyeon Woo Park, Young Ran Kim, Ji Hyang Kim, Woo Sik Lee, Nam Keun Kim

**Affiliations:** 1 Department of Obstetrics and Gynecology, CHA Gangnam Medical Center, CHA University, Seoul 06135, Korea; hycho.md@cha.ac.kr; 2 Department of Biomedical Science, College of Life Science, CHA University, Seongnam 13488, Korea; hahnsung@naver.com (H.S.P.); ejko05@naver.com (E.J.K.); aabb1114@naver.com (H.W.P.); 3 Department of Obstetrics and Gynecology, CHA Bundang Medical Center, CHA University, Seongnam 13496, Korea; bestob@chamc.co.kr (E.H.A.); happyiran@cha.ac.kr (Y.R.K.); bin0902@chamc.co.kr (J.H.K.); 4 Fertility Center of CHA Gangnam Medical Center, CHA University, Seoul 06135, Korea

**Keywords:** plasminogen activator inhibitor-1, tissue plasminogen activator, renin, polymorphism, recurrent pregnancy loss

## Abstract

Recurrent pregnancy loss (RPL) is defined as two or more consecutive pregnancy losses prior to 20 weeks of gestational age. Various factors, including immune dysfunction, endocrine disorders, coagulation abnormality, and genetic disorders influence RPL. In particular, plasminogen activator inhibitor-1 (*PAI-1*), tissue plasminogen activator (*tPA*), and renin (*REN*) have important roles in the thrombotic and thrombolytic systems, and abnormal expression of these genes have a reported negative correlation with pregnancy maintenance. Moreover, some polymorphisms of the three genes are related to expression levels and thrombotic disorder. Therefore, we investigated whether polymorphisms of *PAI-1*, *tPA*, and *REN* are linked to RPL. Genotyping of the six polymorphisms (*PAI-1* rs11178, rs1050955, *tPA* rs4646972, rs2020918, *REN* rs1464816, and rs5707) was performed using polymerase chain reaction (PCR)-restriction fragment length polymorphism and associations of the polymorphisms with RPL were evaluated by statistical analysis. The polymorphism *PAI-1* rs1050955 GA+AA was associated with decreased RPL risk (AOR, 0.528; 95% CI 0.356–0.781; *p* = 0.001) as was the *REN* 10795 rs5707 GG genotype (AOR, 0.487; 95% CI 0.301–0.787; *p* = 0.003). In contrast, the *tPA* rs4646972 II genotype correlated with increased RPL risk (AOR, 1.606; 95% CI, 1.047–2.463; *p* = 0.030). This study provides evidence that *tPA* Alu rs4646972 may contribute to the risk of idiopathic RPL, but *PAI-1* 12068 rs1050955 and *REN* 10795 rs5707 are associated with a decreased risk of RPL. Therefore, these alleles may be useful as biomarkers to evaluate the risk of RPL.

## 1. Introduction

About 10–12% of pregnant women experience early miscarriages, and between 1% and 4% of all pregnant women experience recurrent pregnancy loss (RPL) [1]. While the etiologies of RPL are unclear in a majority of RPL patients, many are suspected to have thrombophilic problems [2]. Hypercoagulability, resulting from an increase in coagulation factors, a decrease in blood flow, and a decrease in the activity levels of natural anticoagulants, leads to an increased susceptibility to venous thromboembolism in pregnant women. Hypofibrinolysis and thrombophilia can be risk factors in RPL and infertility [3]. An imbalance in the dynamic equilibrium between fibrin formation and fibrinolysis will result in a prothrombotic state [4] and thrombophilia, with the formation of a microthrombosis at the embryonic implantation site, resulting in implantation failure and RPL [5].

Systemic or local inflammation play an important role for normal embryo implantation and normal pregnancy development [6]. In particular, the homeostatic balance between pro-inflammatory Th1 cytokines including interferon-γ (IFN-γ) and tumor necrosis factor-α (TNF-α) and anti-inflammatory Th2 such as interleukin-10 (IL-10) is very important for successful pregnancy [7]. TNF-α is encoded on chromosome 6 and produced by NK cells, CD4+, CD8+, macrophages, monocytes, mast cells, neutrophils, and endothelial cells. Nuclear transcription factor-κB (NF-κB) regulates gene expressions for cytokines including IL-1, IL-6, IL-8, and TNF-α and cell adhesion molecules such as ICAM-1, VCAM-1, therefore promoting vascular inflammation and contributing the activation of the hemostatic pathway. Dysregulation of TNF-α leads to vascular dysfunction and pro-coagulant statuses that are related to RPL by regulating IL-6, PAI-1 while decreasing the expression of thrombomodulin [8].

*PAI-1*, also known as the SERPINE1 gene, contains nine exons and encodes the primary inhibitor of *t-PA*. The *PAI-1* rs1050955 polymorphism, located at an miRNA binding site of the *PAI-1* gene, may change the expression level of the gene [9]. The gene for plasminogen activator inhibitor-1 (*PAI-1*), encoding a principal inhibitor of tissue plasminogen activator (tPA) and urokinase (uPA), has been mapped to chromosome 7 (7q21.3-22). Increased concentrations of PAI-1, which plays a role in the conversion of plasminogen to the proteolytic enzyme plasmin [10], affect extracellular proteolysis during ovulation and embryo implantation [11]. The balance between PAI-1 and tPA determines the extent of plasminogen activation and fibrin degradation. The *PAI-1* polymorphism -675 ID, 4G/5G is correlated with an increased risk of RPL [5]. Moreover, the *PAI-1* -675 4G/5G and *tPA* Alu polymorphisms have been associated with thrombotic disorders such as RPL [12], strokes [13], and myocardial infarction [14]. The fibrinolytic system is mainly regulated by tPA and its inhibitor PAI-1, but also by the renin-angiotensin system (RAS), which is a major regulator of blood pressure, sodium balance, and urinary excretion of potassium. The renin gene (*REN*), located on chromosome 1 (1q 32-1q 42), includes a sequence for steroid receptors in its promoter region [15]. Polymorphism in *REN* are associated with high blood pressure and heart failure [16] and in pregnancy complications such as preterm delivery, premature rupture of the membrane, and preeclampsia [15,16,17,18]. RAS control the fibrinolytic system through regulation of plasma PAI-1 and tPA, and this controlling may be affected by genetic variations in RAS and fibrinolytic systems [19,20]. Since interactions between the fibrinolytic system and the RAS could affect the risk of RPL, we hypothesized that polymorphisms in *REN*, *PAI-1*, and *tPA* would be associated with the risk of RPL.

## 2. Results

Analysis of the demographic characteristics and laboratory test values for the RPL and control groups revealed significant differences in the gestation age at the time of the termination of pregnancy and the PT between the two groups (Table 1).

The genotype frequencies of the *PAI-1*, *tPA*, and *REN* genes in the RPL and control groups indicated that the *PAI-1* 12068 rs1050955 GA (AOR, 0.529; 95% CI, 0.349–0.802; *p* = 0.003), AA (AOR, 0.527; 95% CI, 0.319–0.869; *p* = 0.012), and GA+AA (AOR, 0.528; 95% CI, 0.356–0.781; *p* = 0.001) genotypes were correlated with a significant decrease in the risk of RPL, as was the *REN* 10795 rs5707 GG genotype (AOR, 0.487; 95% CI, 0.301–0.787; *p* = 0.003; Table 2). In contrast, the *tPA* Alu rs4646972 II genotype was associated with an increased risk of RPL (AOR, 1.606; 95% CI, 1.047–2.463; *p* = 0.030; Table 2). The *PAI-1* 12068 rs1050955 GA + AA genotype was related to a decrease in both subgroups of RPL patients, those with 2 and ≥3 pregnancy losses (*p* = 0.005 and *p* = 0.006) as was the *REN* 10795 rs5707 GG genotype (*p* = 0.025 and *p* = 0.006; Table S1).

In an analysis of genotype combinations, the combination of *PAI-1* 10692/*PAI-1* 12068 CC/GA (AOR, 0.123; 95% CI 0.051–0.296; *p* < 0.0001), CC/AA (AOR, 0.092; 95% CI 0.018–0.464; *p* = 0.004, and CT/AA (AOR, 0.341; 95% CI 0.155–0.752; *p* = 0.008) were associated with a significantly lower risk of RPL (Table 3). In contrast, the risk of RPL was significantly increased for the *tPA* Alu/*tPA* -7351 DD/TT (AOR, 11.369; 95% CI 2.368–54.575; *p* = 0.002), DI/CT (AOR, 2.497; 95% CI 1.278–4.877; *p* = 0.007), or II/CC (AOR, 4.514; 95% CI 2.184–9.327; *p* < 0.0001) combinations. *REN* 6567/*REN* 10795 GG/GG was linked to a significant decrease in the risk of RPL (AOR, 0.351; 95% CI 0.179–0.685; *p* = 0.002). In an analysis of genotype combinations among *PAI-1*, *tPA* and *REN*, the combination of *tPA* -7351/*REN* 10795 GA/GG showed a significant association with decreased risk of RPL (*p* < 0.001; Table S2). We performed an allele combination analysis of *PAI-1*, *tPA,* and *REN* genes (Table S3). A-C-T and A-C-G allele combinations of *PAI-1* 12068/*tPA* -7351/*REN* 10795 were associated with decreased susceptibility to RPL (*p* = 0.021 and *p* = 0.007, respectively).

The polymorphisms in *PAI-1*, *tPA,* and *REN* were correlated with some clinical parameters in the RPL group (Table 4). An increase in PT was associated with *PAI-1* 12068 rs1050955 AA (*p* = 0.001) and *REN* 6567 rs146814 GG genotype (*p* = 0.039), and an increased activated partial thromboplastin time (aPTT) was associated with the *REN* 10795 rs5707 GG genotype (*p* = 0.046; Table 4). The *tPA* Alu rs4646972 (*p* = 0.040) and *tPA* -7351 *rs2020918* (*p* = 0.022) polymorphisms were associated with changes in the percentages of NK cells. The *tPA* -7351 rs2020918 TT genotype was associated with higher levels of homocysteine (*p* = 0.043).

## 3. Discussion

Successful embryo implantation depends on the regulation of coagulation and fibrinolysis to avoid excess fibrin accumulation in placental vessels and intervillous spaces [21,22]. Overexpression of PAI-1 is associated with thrombus formation in various types of blood vessels [23] and adverse pregnancy complications including miscarriage, stillbirth, fetal growth restriction, and placental abruption [24]. We found that in Korean women with a history of RPL, *PAI-1* polymorphisms were associated with idiopathic RPL and with increased plasma concentrations of vascular risk factors such as homocysteine, folate, and urate [25]. Also, chronically elevated PAI-1 levels due to the *PAI-1* -675 4G allele may affect folliculogenesis and induce ischemic damage to the ovary, leading to primary ovarian insufficiency [26]. High levels of PAI-1 and PLT as well as shorter PT and aPTT are associated with hypofibrinolytic status [27,28]. We demonstrated a negative relationship between RPL and *PAI-1* 12068 rs1050955 GA+AA or *REN* 10795 rs5707 GG, which may be hemophilic or hyperfibrinolytic genotypes that prolong the PT and aPTT.

The miRNA, by base-pairing with target mRNAs at the 3′-untranslated region (UTR), leads to translational repression of target genes. The GA+AA genotype in *PAI-1* rs1050955 was associated with a decreased risk of RPL compared with the GG genotype. *PAI-1* rs2227631 and rs1050955 likely modulate different transcriptional levels of PAI-1, but further studies will be needed to better understand the mechanisms that control PAI-1. Since the *PAI-1* 10692 rs11178 polymorphism did not show an association with susceptibility to RPL, this polymorphism may regulate *PAI-1* differently than the rs1050955 polymorphism, even though both polymorphisms are located in the 3′-UTR of *PAI-1.*

The *tPA* Alu (I/D) polymorphism, which is characterized by the presence or absence of a 311-bp Alu retrotransposon in the 8th intron, was more prevalent among RPL patients compared to the control group. Homozygous carriers of the *tPA* Alu polymorphism show an increase in the release of tPA from vascular endothelial cells [29]. The amount of tPA may be an important factor in the early stages of placental development and placental separation from maternal tissue at delivery [30]. An impaired fibrinolytic system could cause recurrent pregnancy loss through a defect in trophoblast development or excess fibrin deposition in early placentation [31]. This impairment of fibrinolysis could result from significantly higher levels of tPA in patients with *tPA* Alu rs4646972 II compared to Alu rs4646972 DD or DI [29]. The free tPA that is released into blood from endothelial cells immediately combines with circulating PAI-1 forming a PAI-1/tPA complex, whose concentrations in plasma correlate strongly with levels of tPA antigen and PAI-1 activity [32]. The importance of the fibrinolytic system in RPL is supported by the work of Magdoud et al. [33] who found that the −675 4G/5G polymorphism in the *PAI-1* promoter (a) induced high *PAI-1* expression levels, (b) suppressed fibrinolysis and promoted thrombosis through inhibition of plasminogen activator, and (c) was associated with an increased risk of RPL. The increased RPL risk that is associated with the *tPA* Alu polymorphism might be explained by enhanced local recombination, which is characteristic of all Alu elements, if the *tPA* Alu polymorphism is in linkage disequilibrium with an unidentified polymorphism in a coding area of the *tPA* gene that directly affects the protein [34]. However, we did not identify an association of any clinical parameters with *tPA* Alu rs4646972 polymorphism.

The GG genotype of *REN* 10975 rs5707 polymorphism was associated with a decreased risk of RPL in women with either 2 or ≥3 pregnancy losses. Since the *REN* gene is an attractive genetic marker candidate for hypertension associated disease, the *REN* rs5707 polymorphism has been studied in the development of coronary artery disease and preeclampsia, but those studies did not reveal a significant relationship [16,35]. Therefore, the association of *REN* rs5707 polymorphism with RPL is more valuable, and further studies to validate the function of the *REN* rs5707 in RPL are needed.

Several gene polymorphisms affect the risk of RPL, such as matrix metalloproteinase genes [36], the methylenetetrahydrofolate reductase (MTHFR) gene [37], complement factors D and H [38], and genome wide methylation analysis [39], reflecting the complicated mechanisms that underlie the development of RPL. Previous studies have investigated the association of high levels of homocysteine and low levels of folate with the risk of RPL [40,41]. Increased levels of homocysteine and/or decreased levels of folate in the blood have been related to reduced expression of the enzyme MTHFR that catalyzes the process of converting 5,10-methylenetetrahydrofolate to 5-methyltetrahydrofolate and is a critical regulatory enzyme involved in folate metabolism. Kim et al. investigated the association of four 3′-UTR of the MTHFR gene with the risk of RPL [37]. They reported that the 2572C>A, 4869C>G, 5488C>T, and 6685T>C *MTHFR* polymorphisms were significantly associated with RPL susceptibility. In particular, *MTHFR* 4869C>G and 5488C>T genotypes were related to elevated homocysteine levels and reduced folate levels in plasma. We have identified an association between the fibrinolytic system and susceptibility to RPL; however, this study has some limitations. First, this study did not reveal a relationship between RPL and clinical factors such as homocysteine, folate, and PLT. Second, the mechanisms by which the *PAI-1*, *tPA*, and *REN* polymorphisms affect the development of RPL are not clear. Third, the study population included only Korean women, so we do not know whether these same gene polymorphism results apply to other populations. Fourth, we cannot rule out the cases where RPL is caused by other causes, such as chromosomal abnormalities, infection, etc., that have not been evaluated despite the tests for other causes of RPL. Future studies with a larger and more heterogeneous population are needed to confirm our understanding of the influence of the fibrinolytic system on the development of RPL.

In conclusion, to the best of our knowledge, this is the first study to evaluate the association of *PAI-1*, *tPA*, and *REN* gene polymorphisms with the susceptibility of Korean women to RPL. Our findings suggest that polymorphisms in *PAI-1*, *tPA*, and *REN* may contribute to RPL and can be developed as biomarkers to evaluate a woman’s susceptibility to RPL.

## 4. Materials and Methods

### 4.1. Subjects

This study included 206 women in the control group and 334 women with RPL at the Infertility Medical Center of CHA Bundang Medical Center. RPL patients had experienced two or more consecutive spontaneous abortions confirmed by human chorionic gonadotropin levels, ultrasonography, and physical examinations. Patients who used alcohol or smoked were excluded from this study. The women in the control group had a history of at least one successful, naturally-conceived pregnancy and no history of pregnancy loss. Hormonal tests were performed to determine hormonal causes of miscarriage such as thyroid disease, hyperprolactinemia, or luteal insufficiency. Anatomical abnormalities associated with RPL were determined by hysteroscopy, hysterosalpingography, computed tomography (CT), or magnetic resonance imaging (MRI). Possible autoimmune diseases as the cause of miscarriage, such as antiphospholipid syndrome or lupus, were evaluated using lupus anticoagulant and anticardiolipin antibodies. The institutional review board in CHA Bundang Medical Center approved this study and written informed consent was obtained from all participants (IRB number: 2010-01-123).

### 4.2. Genotyping

Genomic DNA (gDNA) was extracted from anticoagulated peripheral blood using a G-DEX II Genomic DNA Extraction Kit (Intron Biotechnology, Seongnam, Korea) according to the manufacturer’s instructions. The concentration and purity of gDNA, which were dissolved in 100 μL of DNA rehydration buffer (Intron Biotechnology, Seongnam, Korea), were measured using nanodrop 1000 (Thermo Fisher Scientific, Waltham, MA, USA). The concentration of all gDNA were above 100 ng/μL and the purity of the gDNA ranged from 1.8 to 2.1 at the 260/280 nm ratio and from 1.9 to 2.2 at the 260/230 nm ratio. gDNA samples were diluted in DNA rehydration buffer to a concentration of 100 ng/μL. The genotypes of the six polymorphisms were determined by polymerase chain reaction (PCR)-restriction fragment length polymorphism (RFLP) analysis with 1 μL of gDNA sample. The six primer sets were designed for easy genotype discrimination using RFLP (Table S4). The PCR was performed with 8 negative controls using distilled water instead of DNA samples per 96 tubes. The *PAI-1* 10692 rs11178 polymorphism was detected using the forward primer (5′-AGA TCT GTC TCC AAG ACC TTG-3′) and the reverse primer (5′-ACA GTG GAC TCT GAG ATG AAA-3′). The *PAI-1* 12068 rs1050955 polymorphism was evaluated by PCR-RFLP analysis using the forward primer (5′-CTA ATA GAA GCC TAA TCA GCC C-3′) and the reverse primer (5′-GTG TGA AAT GGA GAA GGT GAA-3′). The rs4646972 *t-PA* polymorphism, resulting from the presence or absence of an Alu repeat between exon 8 and 9, was determined by the size of the PCR fragment amplified by the primer set (forward: 5′-GTC CTG GCC TGT AAC CAT TTA G-3′ and reverse: 5′-GGA GAC TCA GTC AAC CAA TGA A-3′). The *tPA* -7351 rs2020918 polymorphism was detected with the forward primer (5′-TAA CCA GAA CTG ATG CAA GAT C-3′) and the reverse primer (5′-AAT TTG AGG TTG CAG TGA ACT G-3′). The forward primer for amplification of *REN* 6567 rs1464816 was (5′-CAG AAA TCG GGG TAA GAG TAA-3′), and the reverse primer was (5′-CCC TTC CTT TTT CTG TGA ACT-3′). To detect the *REN* 10795 rs5707 polymorphism, PCR-RFLP analysis was performed with forward (5′-TAA GCT AAC CAG CCA TAC CC-3′) and reverse (5′-AGA GTA GGG TGT TCC TCA GCT-3′) primers. The PCR conditions for amplification of six polymorphism regions were: denaturation (95 °C for 15 min); 30 cycles of denaturation (95 °C for 30 s), annealing (for 30 s), and extension (72 °C for 30 s); and final extension (72 °C for 5 min). The annealing temperature for the two polymorphisms in *tPA* was 58 °C and for the other polymorphisms was 55 °C. For RFLP analysis, the PCR products of *PAI-1* 10692, *PAI-1* 12068, *tPA* -7351, *REN* 6567, and *REN* 10795 were digested with *Bts*CI, *Bst*Z17I, *Ban*II, *Mlu*CI, and *Ava*I, respectively, for 16 h at the recommended temperature. The enzyme was inactivated, and the DNA bands were visualized by electrophoresis in a 3% agarose gel containing DNA staining solution. The RFLP patterns of each polymorphism are presented in Table S4. For each polymorphism, 30% of the PCR analyses were randomly selected and repeated for validation of the PCR-RFLP assay.

### 4.3. Assessment of Clinical Characteristics and Blood Coagulation Status from Blood Samples

Plasma homocysteine, folate, platelet (PLT), activated partial thromboplastin (aPTT), natural killer (NK) cell, prothrombin time (PT), uric acid, total cholesterol, PAI-1, and vascular endothelial growth factor (VEGF) levels were determined in RPL patients after 12 h of fasting. Homocysteine was determined by a fluorescence polarization immunoassay with the Abbott IMx analyzer (Abbott Laboratories, Abbott Park, IL, USA). Folate levels were determined by a competitive immunoassay with ACS:180 (Bayer Diagnostics, Tarrytown, NY, USA). The uric acid and total cholesterol levels were measured with commercially available enzymatic colorimetric tests using the Roche/Hitachi Modular Pre-analytics Plus system (Roche Diagnostics, Mannheim, Germany). PLT, aPTT, PT, and PAI-1 were determined as described previously [36].

### 4.4. Measurement of Peripheral NK Cell Proportions

Estimations of NK cells were determined by flow cytometry with CellQuest software (BD FACSCalibur; BD Biosciences, Seoul, Korea). Fluorescently labeled (fluorescein isothiocyanate, phycoerythrin [PE], peridinin chlorophyll protein, and allophycocyanin) monoclonal antibodies specific for CD3, CD16, and CD56 were purchased from BD Biosciences. Anti-NKG2A-PE antibodies were obtained from Immunotech (Beckman Coulter, Fullerton, CA, USA). All antibodies were used in 1:1000 dilution. Peripheral blood mononuclear cells (2.5 × 10^5^) were stained for cell-surface antigen expression at 4 °C in the dark for 30 min, washed twice in 2 mL of phosphate-buffered saline containing 1% bovine serum albumin and 0.01% sodium azide (FACS wash buffer) and subsequently fixed in 200 μL of a 1% formaldehyde solution (Sigma-Aldrich, St. Louis, MO, USA) before sorting.

### 4.5. Data Analysis

The two-sample t-test and the Mann–Whitney U test were used for the clinical profiles. The associations between *PAI-1*, *t-PA*, and *REN* gene polymorphisms and RPL susceptibility were determined by age-adjusted odds ratios (AOR) and 95% confidence intervals (CIs). Allele frequencies were determined to confirm deviations from the Hardy–Weinberg equilibrium. The allele combinations for the polymorphisms were estimated by the chi-square test and were adjusted using the false discovery rate correction. The association between each *PAI-1, t-PA,* and *REN* gene polymorphism and each clinical value (homocysteine, folate, PLT, aPTT, NK cell, PT, uric acid, total cholesterol, PAI-1, and VEGF) for the RPL patients were assessed by ANOVA and Kruskal–Wallis tests. We considered the *p* < 0.05 of all statistic tests as statistically significant. Analyses were performed using GraphPad Prism 4.0 (GraphPad Software Inc., San Diego, CA, USA) and Medcalc version 12.7.1.0 (Medcalc Software, Mariakerke, Belgium).

## Figures and Tables

**Table 1:** Clinical profiles between RPL patients and control subjects.

Characteristics	Controls (*n* = 206)	RPL Patients (*n* = 334)	*p*
Age (years, mean ± SD)	33.44 ± 5.97	33.4 ± 4.38	0.362
BMI (kg/m^2^, mean ± SD)	21.92 ± 3.47	21.55 ± 3.94	0.415
Previous pregnancy losses (n, mean ± SD)	0	3.06 ± 1.57	
Live birth (*n*, mean ± SD)	1.72 ± 0.72	None	
Average gestational weeks (mean ± SD)	39.37 ± 1.67	7.4 ± 1.95	<0.0001
CD56^+^ NK cells (%, mean ± SD)	None	17.87 ± 8.0	
Homocysteine (μmol/L, mean ± SD)	None	6.92 ± 2.0	
Folate (ng/mL, mean ± SD)	None	14 ± 11.9	
Total cholesterol (mg/dL, mean ± SD)	None	188.73 ± 49.83	
Uric acid (mg/dL, mean ± SD)	None	3.8 ± 0.84	
PLT (10^3^/μL, mean ± SD)	237.36 ± 67.63	254.98 ± 59.36	0.085
aPTT (s, mean ± SD)	33.31 ± 3.84	32.15 ± 4.31	0.307
PT (s, mean ± SD)	11.67 ± 3.25	11.57 ± 0.84	<0.0001

Note: RPL, recurrent pregnancy loss; BMI, body mass index; PLT, platelet count; aPTT, activated partial thromboplastin time; PT, prothrombin time; None, not investigated.

**Table 2:** Genotype frequencies of *PAI-1*, *t-PA* and *REN* gene polymorphisms between controls and RPL patients.

Genotypes	Controls (*n* = 206)	RPL (*n* = 334)	COR (95% CI)	*p*	FDR-*P* ^a^	AOR (95% CI) ^b^	*p*	FDR-*p* ^a^
*PAI-1* 10692 rs11178								
TT	42 (20.4)	69 (20.7)	1.000 (reference)			1.000 (reference)		
TC	102 (49.5)	183 (54.8)	1.092 (0.694–1.719)	0.704	0.824	1.100 (0.698–1.732)	0.683	0.820
CC	62 (30.1)	82 (24.6)	0.805 (0.485–1.335)	0.401	0.474	0.814 (0.490–1.352)	0.427	0.476
Dominant (TT vs. TC+CC)			0.984 (0.640–1.512)	0.940	0.968	0.983 (0.640–1.512)	0.939	0.969
Recessive (TT+TC vs. CC)			0.756 (0.513–1.114)	0.157	0.314	0.756 (0.512–1.115)	0.158	0.316
HWE-*p*	0.997	0.075						
*PAI-1* 12068 rs1050955								
GG	48 (23.3)	122 (36.5)	1.000 (reference)			1.000 (reference)		
GA	109 (52.9)	147 (44.0)	0.531 (0.350–0.804)	0.003	0.018	0.529 (0.349–0.802)	0.003	0.018
AA	49 (23.8)	65 (19.5)	0.522 (0.317–0.860)	0.011	0.033	0.527 (0.319–0.869)	0.012	0.036
Dominant (GG vs. GA+AA)			0.528 (0.357–0.782)	0.001	0.006	0.528 (0.356–0.781)	0.001	0.006
Recessive (GG+GA vs. AA)			0.774 (0.509–1.178)	0.232	0.348	0.773 (0.507–1.177)	0.230	0.345
HWE-*p*	0.403	0.088						
*tPA* Alu rs4646972								
DD	55 (26.7)	74 (22.2)	1.000 (reference)			1.000 (reference)		
DI	113 (54.9)	171 (51.2)	1.125 (0.737–1.716)	0.585	0.824	1.126 (0.735–1.727)	0.586	0.820
II	38 (18.4)	89 (26.6)	1.741 (1.039–2.916)	0.035	0.070	1.692 (1.007–2.842)	0.047	0.094
Dominant (DD vs. DI+II)			1.280 (0.856–1.914)	0.230	0.460	1.282 (0.855–1.924)	0.230	0.460
Recessive (DD+DI vs. II)			1.606 (1.047–2.463)	0.030	0.090	1.606 (1.047–2.463)	0.030	0.090
HWE-*p*	0.133	0.634						
*tPA* -7351 rs2020918								
CC	112 (54.4)	181 (54.2)	1.000 (reference)			1.000 (reference)		
CT	80 (38.8)	124 (37.1)	0.959 (0.665–1.384)	0.824	0.824	0.960 (0.665–1.386)	0.829	0.829
TT	14 (6.8)	29 (8.7)	1.282 (0.649–2.530)	0.474	0.474	1.281 (0.649–2.529)	0.476	0.476
Dominant (CC vs. CT+TT)			1.007 (0.711–1.427)	0.968	0.968	1.007 (0.711–1.427)	0.969	0.969
Recessive (CC+CT vs. TT)			1.304 (0.672–2.530)	0.433	0.458	1.304 (0.672–2.530)	0.433	0.459
HWE-*p*	0.955	0.246						
*REN* 6567 rs1464816								
GG	138 (67.0)	214 (64.1)	1.000 (reference)			1.000 (reference)		
GT	65 (31.6)	112 (33.5)	1.111 (0.765–1.614)	0.580	0.824	1.111 (0.764–1.616)	0.581	0.820
TT	3 (1.5)	8 (2.4)	1.720 (0.448–6.594)	0.429	0.474	1.750 (0.453–6.764)	0.417	0.476
Dominant (GG vs. GT+TT)			1.138 (0.789–1.642)	0.489	0.734	1.138 (0.788–1.645)	0.491	0.737
Recessive (GG+GT vs. TT)			1.661 (0.436–6.332)	0.458	0.458	1.661 (0.434–6.351)	0.459	0.459
HWE-*p*	0.128	0.132						
*REN* 10795 rs5707								
TT	69 (33.5)	137 (41.0)	1.000 (reference)			1.000 (reference)		
TG	95 (46.1)	160 (47.9)	0.848 (0.577–1.247)	0.402	0.824	0.846 (0.575–1.243)	0.393	0.820
GG	42 (20.4)	37 (11.1)	0.444 (0.262–0.753)	0.003	0.018	0.443 (0.261–0.751)	0.003	0.018
Dominant (TT vs. TG+GG)			0.724 (0.504–1.040)	0.081	0.243	0.724 (0.504–1.040)	0.080	0.240
Recessive (TT+TG vs. GG)			0.487 (0.301–0.787)	0.003	0.018	0.487 (0.301–0.787)	0.003	0.018
HWE-*p*	0.377	0.338						

Note: RPL, recurrent pregnancy loss; COR, crude odds ratio; AOR, adjusted odds ratio; CI, confidence interval; FDR, false discovery rate; HWE, Hardy–Weinberg equilibrium. ^a^ FDR-adjusted *p* value; ^b^ Adjusted by age.

**Table 3:** Combination analysis of *PAI-1*, *t-PA* and *REN* gene polymorphisms between RPL patients and controls.

Genotypes	Control (*n* = 206)	RPL (*n* = 334)	AOR (95% CI) ^a^	*p*	FDR-*p* ^b^
*PAI-1* 10692/*PAI-1* 12068					
CC/GG	26 (12.6)	71 (21.3)	1.000 (reference)		
CC/GA	28 (13.6)	9 (2.7)	0.123 (0.051–0.296)	<0.0001	0.0008
CC/AA	8 (3.9)	2 (0.6)	0.092 (0.018–0.464)	0.004	0.016
CT/GG	19 (9.2)	47 (14.1)	0.922 (0.458–1.857)	0.821	0.821
CT/GA	64 (31.1)	118 (35.3)	0.675 (0.393–1.162)	0.156	0.250
CT/AA	19 (9.2)	18 (5.4)	0.341 (0.155–0.752)	0.008	0.021
TT/GG	3 (1.5)	4 (1.2)	0.448 (0.091–2.198)	0.323	0.431
TT/GA	17 (8.3)	20 (6.0)	0.429 (0.195–0.944)	0.035	0.070
TT/AA	22 (10.7)	45 (13.5)	0.758 (0.383–1.499)	0.425	0.486
*tPA* Alu/*tPA* -7351					
DD/CC	28 (13.6)	23 (6.9)	1.000 (reference)		
DD/CT	25 (12.1)	33 (9.9)	1.586 (0.739–3.404)	0.237	0.350
DD/TT	2 (1.0)	18 (5.4)	11.369 (2.368–54.575)	0.002	0.008
DI/CC	62 (30.1)	76 (22.8)	1.454 (0.758–2.790)	0.260	0.350
DI/CT	43 (20.9)	85 (25.4)	2.497 (1.278–4.877)	0.007	0.019
DI/TT	8 (3.9)	10 (3.0)	1.478 (0.499–4.381)	0.481	0.481
II/CC	22 (10.7)	82 (24.6)	4.514 (2.184–9.327)	<0.0001	0.001
II/CT	12 (5.8)	6 (1.8)	0.541 (0.170–1.722)	0.299	0.350
II/TT	4 (1.9)	1 (0.3)	0.306 (0.032–2.946)	0.306	0.350
*REN* 6567/*REN* 10795					
GG/TT	45 (21.8)	87 (26.0)	1.000 (reference)		
GG/TG	63 (30.6)	106 (31.7)	0.858 (0.532–1.384)	0.531	0.850
GG/GG	30 (14.6)	21 (6.3)	0.351 (0.179–0.685)	0.002	0.016
GT/TT	23 (11.2)	46 (13.8)	1.004 (0.540–1.868)	0.989	0.989
GT/TG	31 (15.0)	51 (15.3)	0.780 (0.434–1.401)	0.405	0.850
GT/GG	11 (5.3)	15 (4.5)	0.660 (0.274–1.587)	0.353	0.850
TT/TT	1 (0.5)	4 (1.2)	1.478 (0.152–14.360)	0.736	0.981
TT/TG	1 (0.5)	3 (0.9)	1.200 (0.119–12.071)	0.877	0.989
TT/GG	1 (0.5)	1 (0.3)	0.370 (0.022–6.163)	0.488	0.850

Note: RPL, recurrent pregnancy loss; AOR, adjusted odds ratio; CI, confidence interval. ^a^ adjusted by age; ^b^ FDR-adjusted *p* value.

**Table 4:** Differences of various clinical parameters according to *PAI-1*, *t-PA* and *REN* gene polymorphisms in RPL women.

Genotypes	Homocysteine	Folate	PLT	aPTT	NK cell	PT	Uric acid	T.chol	PAI-1	VEGF
	(mmol/L)	(mg/mL)	(10^3^/uL)	(s)	(%)	(s)	(mg/dL)	(mg/dL)	(mg/dL)	(pg/mL)
	Mean ± SD	Mean ± SD	Mean ± SD	Mean ± SD	Mean ± SD	Mean ± SD	Mean ± SD	Mean ± SD	Mean ± SD	Mean ± SD
*PAI-1* 10692 rs11178										
TT	6.84 ± 2.23	13.91 ± 8.18	236.27 ± 51.51	32.99 ± 4.15	17.96 ± 6.74	11.82 ± 0.66	3.63 ± 0.83	191.97 ± 44.42	10.74 ± 5.83	160.24 ± 137.47
TC	6.89 ± 1.87	13.85 ± 10.20	254.53 ± 62.37	32.02 ± 4.39	17.39 ± 8.70	11.54 ± 0.83	3.87 ± 0.84	192.02 ± 54.03	9.77 ± 5.64	175.36 ± 134.73
CC	7.05 ± 2.07	14.38 ± 16.77	270.75 ± 54.56	31.77 ± 4.27	18.68 ± 7.72	11.46 ± 0.95	3.80 ± 0.85	178.65 ± 42.68	11.26 ± 5.35	173.97 ± 121.35
*p* ^a^	0.819	0.965	0.029	0.379	0.762	0.108	0.431	0.340	0.438	0.865
*PAI-1* 12068 rs1050955										
GG	7.00 ± 2.06	14.10 ± 15.38	265.33 ± 55.22	31.84 ± 4.71	18.41 ± 7.19	11.42 ± 0.86	3.91 ± 0.98	193.33 ± 54.90	11.50 ± 5.58	177.22 ± 130.23
GA	6.79 ± 1.77	13.12 ± 7.79	251.81 ± 63.61	31.73 ± 3.66	17.59 ± 8.85	11.52 ± 0.81	3.82 ± 0.72	186.86 ± 49.27	9.60 ± 5.63	161.20 ± 124.02
AA	7.08 ± 2.38	15.93 ± 13.66	242.27 ± 51.86	34.08 ± 4.81	17.54 ± 7.51	12.07 ± 0.69	3.53 ± 0.83	184.39 ± 39.85	10.54 ± 5.43	189.34 ± 152.60
*p* ^a^	0.634	0.449	0.161	0.088 ^b^	0.858	0.001	0.176	0.674	0.259	0.608
*tPA* Alu rs4646972										
DD	6.74 ± 1.76	13.17 ± 7.07	254.00 ± 51.79	32.37 ± 3.75	18.26 ± 9.82	11.71 ± 0.87	3.78 ± 0.85	186.11 ± 46.32	10.09 ± 4.26	167.47 ± 152.80
DI	6.82 ± 1.90	12.54 ± 7.87	257.65 ± 63.01	32.04 ± 4.70	19.21 ± 7.32	11.54 ± 0.85	3.76 ± 0.87	184.89 ± 52.44	10.91 ± 5.92	175.65 ± 115.40
II	7.23 ± 2.31	17.47 ± 18.84	251.06 ± 58.71	32.19 ± 3.98	14.94 ± 7.26	11.53 ± 0.79	3.91 ± 0.80	198.40 ± 47.30	9.65 ± 5.97	167.78 ± 145.10
*p* ^a^	0.297	0.258 ^b^	0.802	0.917	0.040	0.525	0.613	0.333	0.566 ^b^	0.940
*tPA* -7351 rs2020918										
CC	7.04 ± 2.25	15.24 ± 14.89	254.61 ± 60.38	32.04 ± 4.28	16.01 ± 6.62	11.60 ± 0.73	3.88 ± 0.86	193.18 ± 49.98	10.40 ± 5.47	174.97 ± 132.02
CT	6.59 ± 1.53	13.02 ± 8.09	254.01 ± 57.02	32.41 ± 4.41	20.12 ± 8.62	11.51 ± 0.87	3.75 ± 0.85	186.52 ± 52.35	10.82 ± 5.93	159.00 ± 131.86
TT	7.68 ± 1.99	11.37 ± 5.25	264.08 ± 67.87	31.36 ± 4.15	17.16 ± 9.39	11.81 ± 1.45	3.51 ± 0.61	164.82 ± 21.00	7.04 ± 3.01	206.01 ± 130.91
*p* ^a^	0.043	0.293	0.860	0.693	0.022	0.793 ^b^	0.323	0.186	0.295 ^b^	0.474
*REN* 6567 rs1464816										
GG	6.93 ± 2.17	12.99 ± 10.51	258.69 ± 61.62	32.18 ± 4.06	17.17 ± 6.81	11.68 ± 0.82r	3.85 ± 0.88	191.65 ± 52.82	9.95 ± 5.62	160.46 ± 115.74
GT	6.86 ± 1.74	15.01 ± 13.42	247.39 ± 53.02	31.91 ± 4.37	18.23 ± 8.85	11.38 ± 0.82	3.69 ± 0.78	184.06 ± 45.46	10.93 ± 5.69	183.91 ± 147.79
TT	7.64 ± 1.80	16.85 ± 7.83	263.67 ± 99.33	36.23 ± 11.28	21.00 ± 6.16	11.10 ± 1.15	4.23 ± 0.56	177.00 ± 14.76	11.24 ± 0.17	154.40 ± 87.28
*p* ^a^	0.690	0.440	0.453	0.829 ^b^	0.565	0.039	0.340	0.590	0.627	0.921 ^b^
*REN* 10795 rs5707										
TT	6.76 ± 1.88	13.52 ± 7.87	255.99 ± 62.59	31.40 ± 3.80	18.48 ± 8.62	11.49 ± 0.89	3.72 ± 0.83	182.97 ± 44.32	10.25 ± 5.66	161.94 ± 125.44
TG	6.98 ± 2.15	13.81 ± 10.98	254.80 ± 58.37	32.23 ± 4.02	18.21 ± 7.48	11.58 ± 0.76	3.85 ± 0.88	196.58 ± 55.91	10.53 ± 5.34	168.21 ± 126.44
GG	7.26 ± 1.74	16.94 ± 24.46	251.58 ± 52.27	34.60 ± 6.21	13.61 ± 5.99	11.86 ± 0.90	3.96 ± 0.71	174.44 ± 33.05	10.20 ± 6.58	216.56 ± 163.85
*p* ^a^	0.454	0.486	0.958	0.046 ^b^	0.146	0.186	0.480	0.395 ^b^	0.959	0.292

Note: PLT, platelet count; aPTT, activated partial thromboplastin time; NK cell, natural killer cell; PT, prothrombin time; T.chol, total cholesterol; PAI-1, plasminogen activator inhibitor-1; VEGF, vascular endothelial growth factor. ^a^ ANOVA; ^b^ Kruskal–Wallis test.

## Data Availability

Data sharing not applicable.

## Supplementary Materials

The following are available online at https://www.mdpi.com/article/10.3390/jpm11121378/s1, Table S1: Genotype frequencies of PAI-1, t-PA and REN gene polymorphisms according to the number of RPL; Table S2: Genotype combination analysis of PAI-1, t-PA and REN gene polymorphisms between RPL patients and controls; Table S3: Allele combination analysis of PAI-1, tPA and REN gene polymorphisms in RPL and controls; Table S4: Information for PCR and RFLP analysis of each variants.
