# Supplementary material for: Association of Polymorphisms in Plasminogen Activator Inhibitor-1 (PAI-1), Tissue Plasminogen Activator (tPA), and Renin (REN) with Recurrent Pregnancy Loss in Korean Women
Source: J Pers Med. 2021 Dec 16;11(12):1378. doi: 10.3390/jpm11121378 (PMC8705673; doi:10.3390/jpm11121378)
Supplement: Supplementary file 1 [file jpm-11-01378-s001.zip › jpm-1476331-supplementary.pdf]

Supplementary Table S1. Genotype frequencies of *PAI-1*, *t-PA* and *REN* gene polymorphisms according to the number of RPL.

Genotypes	Controls (n=206)	PL=2 (n=154)	AOR (95% CI)[a]	*p*	FDR-*p*[b]	PL≥3 (n=180)	AOR (95% CI)[a]	*p*	FDR-*p*[b]
PAI-1 10692 rs11178									
TT	42 (20.4)	33 (21.4)	1.000 (reference)			36 (20.0)	1.000 (reference)		
TC	102 (49.5)	85 (55.2)	1.062 (0.619 - 1.824)	0.826	0.922	98 (54.4)	1.135 (0.670 - 1.922)	0.637	0.748
CC	62 (30.1)	36 (23.4)	0.750 (0.405 - 1.391)	0.362	0.443	46 (25.6)	0.876 (0.486 - 1.576)	0.658	0.658
Dominant (TT vs. TC+CC)			0.934 (0.559 - 1.561)	0.794	0.939		1.025 (0.623 - 1.688)	0.922	0.922
Recessive (TT+TC vs. CC)			0.718 (0.445 - 1.159)	0.175	0.263		0.789 (0.503 - 1.236)	0.301	0.602
PAI-1 12068 rs1050955									
GG	48 (23.3)	57 (37.0)	1.000 (reference)			65 (36.1)	1.000 (reference)		
GA	109 (52.9)	71 (46.1)	0.548 (0.337 - 0.891)	0.015	0.090	76 (42.2)	0.514 (0.319 - 0.826)	0.006	0.036
AA	49 (23.8)	26 (16.9)	0.457 (0.247 - 0.844)	0.012	0.072	39 (21.7)	0.584 (0.332 - 1.029)	0.063	0.176
Dominant (GG vs. GA+AA)			0.519 (0.328 - 0.823)	0.005	0.030		0.534 (0.342 - 0.833)	0.006	0.036
Recessive (GG+GA vs. AA)			0.657 (0.386 - 1.118)	0.121	0.242		0.871 (0.538 - 1.408)	0.572	0.689
tPA Alu rs4646972									
DD	55 (26.7)	36 (23.4)	1.000 (reference)			38 (21.1)	1.000 (reference)		
DI	113 (54.9)	74 (48.1)	0.974 (0.578 - 1.644)	0.922	0.922	97 (53.9)	1.274 (0.770 - 2.110)	0.346	0.692
II	38 (18.4)	44 (28.6)	1.670 (0.906 - 3.079)	0.100	0.200	45 (25.0)	1.688 (0.926 - 3.075)	0.088	0.176
Dominant (DD vs. DI+II)			1.163 (0.712 - 1.900)	0.548	0.939		1.398 (0.866 - 2.255)	0.170	0.346
Recessive (DD+DI vs. II)			1.765 (1.074 - 2.900)	0.025	0.135		1.469 (0.902 - 2.394)	0.122	0.366
tPA -7351 rs2020918									
CC	112 (54.4)	87 (56.5)	1.000 (reference)			94 (52.2)	1.000 (reference)		
CT	80 (38.8)	52 (33.8)	0.842 (0.537 - 1.318)	0.451	0.902	72 (40.0)	1.071 (0.704 - 1.631)	0.748	0.748
TT	14 (6.8)	15 (9.7)	1.379 (0.632 - 3.010)	0.419	0.443	14 (7.8)	1.220 (0.552 - 2.695)	0.623	0.658
Dominant (CC vs. CT+TT)			0.920 (0.604 - 1.401)	0.698	0.939		1.090 (0.730 - 1.628)	0.673	0.808
Recessive (CC+CT vs. TT)			1.476 (0.690 - 3.158)	0.316	0.379		1.170 (0.542 - 2.528)	0.689	0.689
REN 6567 rs1464816									
GG	138 (67.0)	104 (67.5)	1.000 (reference)			110 (61.1)	1.000 (reference)		
GT	65 (31.6)	46 (29.9)	0.944 (0.599 - 1.490)	0.805	0.922	66 (36.7)	1.259 (0.821 - 1.929)	0.291	0.692
TT	3 (1.5)	4 (2.6)	1.818 (0.395 - 8.362)	0.443	0.443	4 (2.2)	1.683 (0.363 - 7.799)	0.506	0.658
Dominant (GG vs. GT+TT)			0.983 (0.629 - 1.535)	0.939	0.939		1.274 (0.837 - 1.940)	0.259	0.389
Recessive (GG+GT vs. TT)			1.864 (0.410 - 8.482)	0.420	0.420		1.479 (0.325 - 6.734)	0.613	0.689
REN 10795 rs5707									
TT	69 (33.5)	65 (42.2)	1.000 (reference)			72 (40.0)	1.000 (reference)		
TG	95 (46.1)	70 (45.5)	0.783 (0.495 - 1.239)	0.296	0.888	90 (50.0)	0.900 (0.580 - 1.397)	0.638	0.748
GG	42 (20.4)	19 (12.3)	0.481 (0.254 - 0.911)	0.025	0.075	18 (10.0)	0.400 (0.210 - 0.765)	0.006	0.036
Dominant (TT vs. TG+GG)			0.693 (0.450 - 1.068)	0.096	0.288		0.748 (0.493 - 1.135)	0.173	0.346
Recessive (TT+TG vs. GG)			0.548 (0.304 - 0.987)	0.045	0.135		0.431 (0.238 - 0.781)	0.006	0.036

Note: RPL, recurrent pregnancy loss; PL, pregnancy loss; AOR, adjusted odds ratio; CI, confidence interval; FDR, false discovery rate. [a] Adjusted by age; [b] FDR-adjusted *p* value.

Supplementary Table S2. Genotype combination analysis of *PAI-1*, *t-PA* and *REN* gene polymorphisms between RPL patients and controls.

Genotype	Control (n= 206)	RPL (n=334)	AOR (95% CI)[a]	*P*	FDR-*P*[b]
PAI-1 10692/*tPA* -7351					
CC/CC	38 (18.4)	41 (12.3)	1.000 (reference)		
CC/CT	20 (9.7)	35 (10.5)	1.622 (0.796 - 3.304)	0.183	0.394
CC/TT	4 (1.9)	6 (1.8)	1.430 (0.372 - 5.492)	0.602	0.688
CT/CC	50 (24.3)	101 (30.2)	1.861 (1.065 - 3.251)	0.029	0.212
CT/CT	46 (22.3)	65 (19.5)	1.291 (0.719 - 2.319)	0.393	0.524
CT/TT	6 (2.9)	17 (5.1)	2.824 (0.987 - 8.084)	0.053	0.212
TT/CC	24 (11.7)	39 (11.7)	1.474 (0.748 - 2.903)	0.263	0.421
TT/CT	14 (6.8)	24 (7.2)	1.709 (0.758 - 3.855)	0.197	0.394
TT/TT	4 (1.9)	6 (1.8)	1.250 (0.322 - 4.850)	0.747	0.747
PAI-1 12068/*tPA* -7351					
GG/CC	25 (12.1)	68 (20.4)	1.000 (reference)		
GG/CT	19 (9.2)	45 (13.5)	0.871 (0.430 - 1.767)	0.702	0.766
GG/TT	4 (1.9)	9 (2.7)	0.825 (0.233 - 2.924)	0.766	0.766
GA/CC	59 (28.6)	73 (21.9)	0.456 (0.257 - 0.809)	0.007	0.056
GA/CT	45 (21.8)	58 (17.4)	0.470 (0.257 - 0.859)	0.014	0.056
GA/TT	5 (2.4)	16 (4.8)	1.415 (0.453 - 4.418)	0.551	0.735
AA/CC	28 (13.6)	40 (12.0)	0.545 (0.278 - 1.066)	0.076	0.131
AA/CT	16 (7.8)	21 (6.3)	0.490 (0.220 - 1.095)	0.082	0.131
AA/TT	5 (2.4)	4 (1.2)	0.249 (0.060 - 1.029)	0.055	0.131
PAI-1 10692/*REN* 10795					
CC/TT	25 (12.1)	53 (15.9)	1.000 (reference)		
CC/TG	39 (18.9)	52 (15.6)	0.628 (0.332 - 1.188)	0.152	0.405
CC/GG	16 (7.8)	14 (4.2)	0.415 (0.175 - 0.981)	0.045	0.180
CT/TT	38 (18.4)	67 (20.1)	0.834 (0.448 - 1.551)	0.566	0.675
CT/TG	42 (20.4)	87 (26.0)	0.958 (0.523 - 1.755)	0.890	0.890
CT/GG	21 (10.2)	16 (4.8)	0.359 (0.160 - 0.804)	0.013	0.104
TT/TT	6 (2.9)	17 (5.1)	1.333 (0.468 - 3.800)	0.591	0.675
TT/TG	14 (6.8)	21 (6.3)	0.712 (0.311 - 1.629)	0.421	0.675
TT/GG	5 (2.4)	7 (2.1)	0.646 (0.186 - 2.251)	0.493	0.675
PAI-1 12068/*REN* 6567					
GG/GG	29 (14.1)	75 (22.5)	1.000 (reference)		
GG/GT	17 (8.3)	42 (12.6)	0.947 (0.466 - 1.924)	0.880	0.995
GG/TT	2 (1.0)	5 (1.5)	0.937 (0.169 - 5.201)	0.941	0.995
GA/GG	76 (36.9)	100 (29.9)	0.508 (0.301 - 0.857)	0.011	0.060
GA/GT	33 (16.0)	46 (13.8)	0.522 (0.279 - 0.976)	0.042	0.112
GA/TT	0 (0.0)	1 (0.3)	NA	0.995	0.995
AA/GG	33 (16.0)	39 (11.7)	0.456 (0.243 - 0.858)	0.015	0.060
AA/GT	15 (7.3)	24 (7.2)	0.733 (0.326 - 1.649)	0.452	0.904
AA/TT	1 (0.5)	2 (0.6)	0.754 (0.064 - 8.819)	0.822	0.995
PAI-1 12068/*REN* 10795					
GG/TT	18 (8.7)	52 (15.6)	1.000 (reference)		
GG/TG	24 (11.7)	53 (15.9)	0.769 (0.373 - 1.585)	0.477	0.545
GG/GG	6 (2.9)	17 (5.1)	0.988 (0.336 - 2.900)	0.982	0.982
GA/TT	32 (15.5)	57 (17.1)	0.632 (0.317 - 1.264)	0.195	0.276
GA/TG	48 (23.3)	80 (24.0)	0.573 (0.300 - 1.095)	0.092	0.192
GA/GG	29 (14.1)	10 (3.0)	0.119 (0.049 - 0.292)	<0.0001	0.0008

AA/TT	19 (9.2)	28 (8.4)	0.509 (0.230 - 1.126)	0.096	0.192
AA/TG	23 (11.2)	27 (8.1)	0.417 (0.192 - 0.907)	0.027	0.108
AA/GG	7 (3.4)	10 (3.0)	0.490 (0.162 - 1.486)	0.207	0.276
tPA -7351/*REN* 10795					
CC/TT	38 (18.4)	72 (21.6)	1.000 (reference)		
CC/TG	51 (24.8)	85 (25.4)	0.881 (0.521 - 1.489)	0.636	0.727
CC/GG	23 (11.2)	24 (7.2)	0.551 (0.275 - 1.104)	0.093	0.372
CT/TT	25 (12.1)	57 (17.1)	1.207 (0.653 - 2.232)	0.548	0.727
CT/TG	37 (18.0)	56 (16.8)	0.800 (0.451 - 1.418)	0.445	0.727
CT/GG	18 (8.7)	11 (3.3)	0.321 (0.138 - 0.751)	0.009	0.072
TT/TT	6 (2.9)	8 (2.4)	0.705 (0.228 - 2.180)	0.543	0.727
TT/TG	7 (3.4)	19 (5.7)	1.440 (0.553 - 3.747)	0.455	0.727
TT/GG	1 (0.5)	2 (0.6)	1.052 (0.092 - 11.985)	0.967	0.967

Note: RPL, recurrent pregnancy loss; AOR, adjusted odds ratio; CI, confidence interval; FDR, false discovery rate. [a] Adjusted by age; [b] FDR-adjusted *p* value.

Supplementary Table S3. Allele combination analysis of *PAI-1*, *tPA* and *REN* gene polymorphisms in RPL and controls.

Allele combinations	Controls (2n=412)	RPL (2n=668)	OR (95% CI)	p^a	FDR-p^b
tPA Alu/*tPA* -7351					
D-C	294 (71.3)	435 (65.1)	1.000 (reference)		
D-T	24 (5.7)	59 (8.8)	1.661 (1.011 - 2.732)	0.044	0.066
I-C	64 (15.6)	142 (21.3)	1.500 (1.078 - 2.086)	0.018	0.054
I-T	30 (7.4)	32 (4.8)	0.721 (0.429 - 1.212)	0.228	0.228
PAI-1 12068/*tPA* -7351/*REN* 10795					
G-C-T	253 (61.4)	450 (67.4)	1.000 (reference)		
G-C-G	32 (7.8)	40 (6.1)	0.703 (0.431 - 1.147)	0.160	0.373
G-T-T	16 (3.8)	23 (3.5)	0.808 (0.419 - 1.558)	0.608	0.906
G-T-G	8 (1.9)	15 (2.3)	1.054 (0.441 - 2.521)	1.000	1.000
A-C-T	20 (4.9)	13 (1.9)	0.365 (0.179 - 0.747)	0.006	0.021
A-C-G	14 (3.4)	4 (0.6)	0.161 (0.052 - 0.493)	0.001	0.007
A-T-T	34 (8.2)	65 (9.7)	1.075 (0.690 - 1.673)	0.823	0.960
A-T-G	36 (8.7)	57 (8.5)	0.890 (0.571 - 1.389)	0.647	0.906
PAI-1 12068/*PAI-1* 10692/*tPA* Alu/*tPA* -7351					
T-G-D-C	250 (60.6)	392 (58.7)	1.000 (reference)		
T-G-D-T	8 (2.0)	25 (3.8)	1.993 (0.885 - 4.489)	0.100	0.300
T-G-I-C	22 (5.4)	61 (9.2)	1.768 (1.059 - 2.953)	0.030	0.113
T-G-I-T	5 (1.3)	12 (1.8)	1.531 (0.533 - 4.398)	0.615	0.776
T-A-D-C	17 (4.2)	10 (1.5)	0.375 (0.169 - 0.833)	0.016	0.113
T-A-D-T	2 (0.6)	3 (0.4)	0.957 (0.159 - 5.768)	1.000	1.000
T-A-I-C	10 (2.5)	4 (0.6)	0.255 (0.079 - 0.822)	0.024	0.113
T-A-I-T	4 (0.9)	0 (0.0)	0.071 (0.004 - 1.324)	0.024	0.113
C-G-D-C	12 (2.9)	13 (1.9)	0.691 (0.310 - 1.539)	0.406	0.620
C-G-D-T	1 (0.2)	5 (0.8)	3.189 (0.370 - 27.470)	0.413	0.620
C-G-I-C	6 (1.4)	17 (2.5)	1.807 (0.703 - 4.646)	0.277	0.594
C-G-I-T	5 (1.2)	4 (0.6)	0.510 (0.136 - 1.919)	0.324	0.608
C-A-D-C	17 (4.2)	26 (3.9)	0.975 (0.519 - 1.835)	1.000	1.000
C-A-D-T	10 (2.5)	19 (2.8)	1.212 (0.554 - 2.649)	0.700	0.808
C-A-I-C	24 (5.8)	54 (8.0)	1.435 (0.865 - 2.381)	0.176	0.440
C-A-I-T	18 (4.5)	23 (3.5)	0.815 (0.431 - 1.541)	0.621	0.776

Note: RPL, recurrent pregnancy loss; OR, odds ratio; CI, confidence interval; FDR, false discovery rate. [a] Fisher's exact test; [b] FDR-adjusted *p* value.

Supplementary Table S4. Information for PCR and RFLP analysis of each variants.

Variants	Primer sequence		Annealing	Enzyme	Enzyme reaction	Heat inactivation	RFLP pattern
PAI-1 10692 rs11178 T>C	Forward:<br>Reverse:	5'- AGA TCT GTC TCC AAG ACC TTG -3'<br>5'- ACA GTG GAC TCT GAG ATG AAA -3'	55˚C	*Bts*CI	50˚C, 16h	80˚C, 20m	TT: 156bp, 223bp<br>CT: 156bp, 223bp, 379bp<br>CC: 379bp
PAI-1 12068 rs1050955 G>A	Forward:<br>Reverse:	5'- CTA ATA GAA GCC TAA TCA GCC C -3'<br>5'- GTG TGA AAT GGA GAA GGT GAA -3'	55˚C	*Bst*Z17I	37˚C, 16h	-	GG: 278bp,<br>GA: 278bp, 163bp, 115bp<br>AA: 163bp, 115bp
tPA Alu rs4646972 D>I	Forward:<br>Reverse:	5'- GTC CTG GCC TGT AAC CAT TTA G -3'<br>5'- GGA GAC TCA GTC AAC CAA TGA A -3'	58˚C	-	-	-	II: 560bp<br>ID: 560bp, 249bp<br>DD: 249bp
tPA -7351 rs2020918 C>T	Forward:<br>Reverse:	5'- TAA CCA GAA CTG ATG CAA GAT C -3'<br>5'- AAT TTG AGG TTG CAG TGA ACT G -3'	58˚C	*Ban*II	37˚C, 16h	80˚C, 20m	CC: 124bp, 196bp<br>CT: 124bp, 196bp, 324bp<br>TT: 324bp
REN 6567 rs1464816 G>T	Forward:<br>Reverse:	5'- CAG AAA TCG GGG TAA GAG TAA-3'<br>5'- CCC TTC CTT TTT CTG TGA ACT -3'	55˚C	*Mlu*CI	37˚C, 16h	-	TT: 286bp, 23bp<br>GT: 309bp, 286bp, 23bp<br>GG: 309bp
REN 10795 rs5707 T>G	Forward:<br>Reverse:	5'- TAA GCT AAC CAG CCA TAC CC -3'<br>5'- AGA GTA GGG TGT TCC TCA GCT -3'	55˚C	*Ava*I	37˚C, 16h	80˚C, 20m	TT: 351bp<br>TG: 351bp, 227bp, 124bp<br>GG: 227bp, 124bp

Note: PCR, polymerase chain reaction; RFLP, restriction fragment length polymorphism
